# Editorial: Post-stroke cognitive decline and dementia: unraveling mechanisms, models, and biomarkers

**DOI:** 10.3389/fstro.2025.1646796

**Published:** 2025-07-11

**Authors:** Robert T. Mallet, Rebecca F. Gottesman, Paco S. Herson

**Affiliations:** 1 Department of Physiology and Anatomy, University of North Texas Health Science Center, Fort Worth, TX, United States,; 2 Intramural Research Program, National Institute of Neurological Disorders and Stroke, National Institutes of Health, Bethesda, MD, United States,; 3 Department of Neurological Surgery, The Ohio State University College of Medicine, Columbus, OH, United States

**Keywords:** biomarkers, cerebrovascular, cognitive impairment, dementia, ischemic stroke, microvascular rarefaction

## Vascular cognitive impairment and its etiologies

By depriving the brain of the O_2_ and energy substrates essential for neuronal function and survival, cerebral ischemia ignites a complex cascade of neuronal injury central to the pathogenesis of vascular cognitive impairment, a class of neurocognitive disorders of diverse and complex etiology frequently culminating in dementia (Wong and Chui, 2022). The articles comprising the Research Topic on post-stroke cognitive decline and dementia examined the pathogenesis and potential biomarkers of two prominent vascular cognitive impairment subtypes, post-stroke cognitive impairment (PSCI) and vascular dementia, in human patients and rodent models of these disorders.

Approximately 70% of stroke victims develop cognitive impairment within 1 year (Rost et al., 2022). Infarct volume and location, comorbidities, age and heredity impact the trajectory of cognitive decline following an acute cerebrovascular event (Guo et al., 2024). Xu et al. assessed the impact of infarct volume on PSCI severity. Larger infarcts augured more severe cognitive impairment; lesions exceeding 0.054 ml discriminated, with good sensitivity and specificity, patients with PSCI from cognitively intact survivors. Thus, infarct volume analysis may inform PSCI prognosis.

Microvascular rarefaction, an insidious process of capillary degeneration that gradually deprives the brain parenchyma of fuel and oxygen, culminates in vascular dementia. White matter hyperintensities (WMH) characterize cerebral small vessel disease and associated blood brain barrier (BBB) disruption and cerebral edema (Inoue et al., 2023). In ischemic stroke patients Okine et al. correlated WMH severity with diffuse BBB disruption detected by gadolinium FLAIR MRI, and elevated NIH stroke score at discharge. These findings corroborate the hypothesis that BBB disruption links cerebral small vessel disease with adverse stroke outcomes.

Functional magnetic resonance imaging (fMRI) offers a non-invasive means of examining interregional communication during higher-order cerebral functions, potentially revealing vascular cognitive impairment. Zhang et al.’s meta-analysis of resting state fMRI studies in cognitively impaired patients revealed altered activities of brain regions involved in memory, language processing and complex cognition. Decreased activities of the bilateral precuneus and medial frontal gyrus were ascribed to cognitive deficits, while decreased regional homogeneity in the right subgyral region and middle temporal gyrus may have contributed to language difficulties. Functional connectivity increased in regions involved in complex cognition, but decreased in memory-associated regions.

Using questionnaires and cognitive testing, Björck et al. identified determinants of successful return to daily life in elderly stroke survivors. Fewer than 20% of survivors with cognitive impairment, fatigue and/or disequilibrium resumed normal daily activities, while survivors without those limitations were severalfold more likely to resume daily living. The survivors’ self-assessments of memory generally exceeded the objective measures, showing comprehensive cognitive testing to be indispensable for evaluating PSCI in stroke survivors.

## Mechanisms of vascular cognitive impairment

Loss of cerebrovascular integrity and chronic inflammation following stroke may accelerate β-amyloid deposition in the cerebral parenchyma (Okamoto et al., 2012). Interaction of β-amyloid with its microglial receptors may promote pro-inflammatory, phagocytic microglial polarization and release of cytokines and chemokines that elicit activated T-cell invasion (Zhao et al., 2018). Khan et al. reviewed evidence linking cerebral artery occlusion with amyloid angiopathy. Studies in mice implicated tau pathology, β-amyloid deposition and chronic brain inflammation in post-stroke cognitive decline. Altered extracellular matrix protein composition could compromise BBB integrity causing microvascular leakage and rarefaction.

Mei et al. hypothesized that intravenous thrombolysis during acute, mild stroke minimizes subsequent cognitive impairment. Cognitive function was evaluated 3–5 days after stroke onset; local blood flow, a measure of regional neuronal activity due to neurovascular coupling (Iadecola, 2017), was analyzed by arterial spin labeling. Multivariate regression analysis identified significant associations between acute thrombolysis and cognitive performance, indicating attenuation of brain network damage by thrombolysis.

The *ecto* enzyme CD38 hydrolyzes NAD^+^, generating ADP-ribose (Rah et al., 2023), a ligand of neuronal TRPM2 Ca^2+^ channels (Perraud et al., 2001). Although essential for synaptogenesis during normal brain development, CD38 is implicated in post-ischemic neurodegeneration (Guerreiro et al., 2020). Burch et al. examined CD38-mediated responses in mice 7 days after cerebral ischemia imposed by cardiac arrest. Increased CD38 mRNA abundance and protein content colocalized with astrocyte marker glial fibrillary acidic protein in the CA1 subregion, while CD38 inhibition preserved hippocampal synaptic plasticity and long-term potentiation. These findings implicate astroglial CD38 activation of TRPM2 Ca^2+^ channels on adjacent neurons, in post-ischemic deficits of neuronal long-term potentiation essential for learning and memory.

In rats modeling vascular dementia, Jiang et al. interrogated the mechanisms whereby Tongqiao Huashuan (TH), a blend of eight medicinal herbs containing antioxidant and anti-inflammatory compounds, preserves spatial learning and memory. Neurons in the hippocampal CA1 subregion, pivotal for memory encoding, consolidation and retrieval (Watarai et al., 2021; Geiller et al., 2023; Vanrobaeys et al., 2023), are exceptionally vulnerable to ischemia (Sadowski et al., 1999; Sharma et al., 2008). A 3-week TH-donepezil regimen prevented accumulation of the autophagy factors BECLIN-1 and LC3, activated neuroprotective PI3K—Akt—mTOR signaling, protected CA1 neurons and preserved learning and memory. Diverse chemical mixtures like TH may interrupt vascular dementia’s multifarious pathogenesis more effectively than monotherapies.

## Biomarkers of vascular cognitive impairment

**FIGURE 1:**
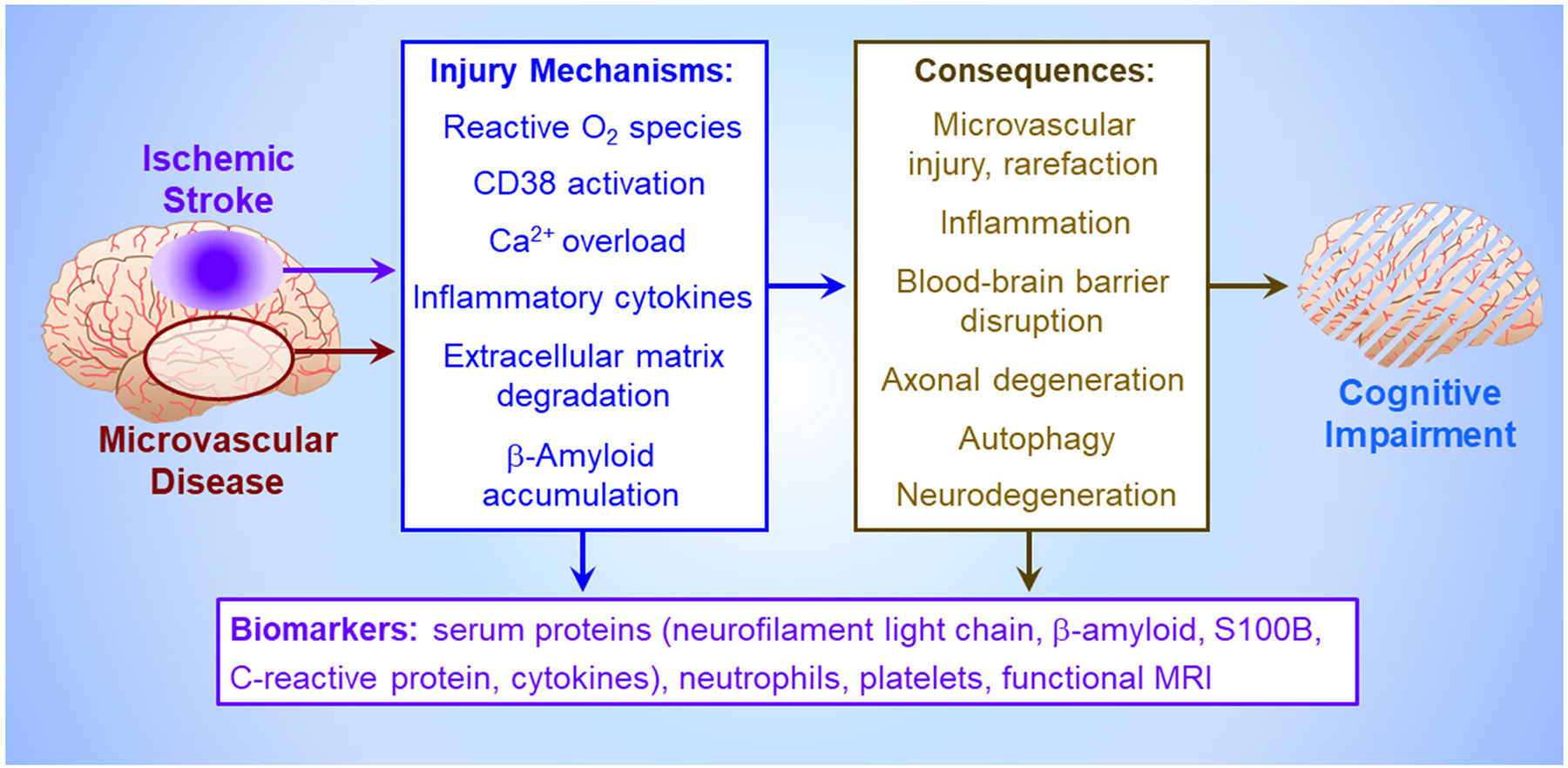
Pathogenesis of vascular cognitive impairment. Ischemia, either imposed abruptly by cerebrovascular occlusion (Mei et al.), or more insidiously by cerebral microvascular disease, triggers an injury crescendo mediated by reactive O_2_ metabolites, microglial release of inflammatory cytokines, neuronal Ca^2+^ channel activation by astroglial CD38 causing neuronal Ca^2+^ overload (Burch et al.), extracellular matrix degradation by matrix metalloproteinases, and/or interstitial β-amyloid deposition (Khan et al.). The consequences of these injury mechanisms, which collectively impair cognitive function (Björck et al.), include death of brain parenchyma in the infarct core (Xu et al.), uncontrolled inflammation and autophagy which harm neurons and astroglia in the peri-infarct penumbra (Jiang et al.), loss of blood brain barrier integrity (Okine et al.), degeneration of axons and entire neurons, and cerebral microvessel rarefaction. These injury cascades generate circulating biomarkers potentially presaging cognitive impairment, including inflammatory factors (Hong et al.), structural proteins from degenerating neurons, circulating platelets and leukocytes (Mao et al.), and β-amyloid and astroglial S100B effluxing across the disrupted blood brain barrier (Huang et al.), culminating in altered local neural activity and interregional connectivity detectable by functional magnetic resonance imaging (Zhang et al.).

Circulating biomarkers (Figure 1) may facilitate early assessment of PSCI risk in stroke survivors. Hong et al.’s network analysis of protein-protein interactions revealed multi-factorial PSCI pathogenesis involving vascular endothelial growth factor, interleukin-6 and C-reactive protein. Comprehensive analysis of vascular injury, inflammation, neuroaxonal injury and biomarkers of neurodegeneration may afford greater prognostic power than single factors.

During acute evolution of brain infarct, platelets and neutrophils secrete factors prompting inflammatory leukocyte infiltration and pro-inflammatory microglial polarization (Kollikowski et al., 2022). Mao et al.’s meta-analysis of high-quality cohort and case-control studies of stroke survivors examined whether lymphocyte- and platelet-associated inflammation indices could predict PSCI. Cognitively impaired survivors had consistently higher neutrophil-to-lymphocyte and platelet-to-lymphocyte ratios than their cognitively normal counterparts. Dynamic changes in these ratios as brain injury evolves are not yet known.

Detection of microvascular rarefaction at its early stages may afford timely treatment. Huang et al. conducted a meta-analysis of clinical studies directed toward identifying serum biomarkers of vascular cognitive impairment. Their analysis revealed increases in neurofilament light chain, a marker of axonal injury (Gaetani et al., 2019), and the astroglial Ca^2+^ binding protein S100B, an indicator of BBB disruption (Roberts et al., 2021), while serum amyloid-β42 (Aβ42) and Aβ42/Aβ40 fell.

## Future directions

Preclinical studies of the later stages of post-stroke pathology are essential to decipher the mechanisms of protracted cognitive decline. PSCI and vascular dementia likely result from complex interactions of vascular damage, neurodegeneration and chronic inflammation (Figure 1). Understanding the temporal evolution of these mechanisms and their interactions is essential to discern the pathogenesis of cognitive impairment.

Development of effective interventions for PSCI and vascular dementia hinges on translational studies bridging preclinical research and clinical practice. Potential therapeutics proven effective in animal models must withstand the scrutiny of clinical trials before their application to cognitively impaired patients. Similarly, randomized, multi-center clinical trials are required to firmly establish circulating factors as prognostic biomarkers of vascular cognitive impairment.

## Abbreviations:

Akt: protein kinase B
BBB: blood-brain barrier
CA1: cornu ammonis-1 subregion
mTOR: mammalian target of rapamycin
PI3K: phosphatidylinositol trisphosphate-dependent protein kinase
PSCI: post-stroke cognitive impairment
rs-fMRI: resting state functional magnetic resonance imaging
TH: Tongqiao Huashuan
TRPM2: transient receptor potential melastatin 2 channel
WMH: white matter hyperintensity
